# Trends in antibiotic use among cardiovascular heart disease inpatients at the Jakaya Kikwete Cardiac Institute in Tanzania from 2016 to 2022

**DOI:** 10.1017/ash.2025.10241

**Published:** 2025-11-25

**Authors:** Jackob Aron Ndayomwami, Judith Ambele Mwamelo, Hamis Abdalla Kaniki, James Mwakyomo, Naizihijwa Majani, Reuben Kato Mutagaywa, Peter Kisenge, Mohamed Janabi, Raphael Zozimus Sangeda

**Affiliations:** 1 Department of Pharmaceutical Microbiology, Muhimbili University of Health and Allied Sciences, Dar es Salaam, Tanzania; 2 Department of Clinical Audit and Quality Assurance, Jakaya Kikwete Cardiac Institute, Dar es Salaam, Tanzania; 3 Department of Internal Medicine, Muhimbili University of Health and Allied Sciences, Dar es Salaam, Tanzania; 4 Department of Adult Cardiology, Jakaya Kikwete Cardiac Institute, Dar es Salaam, Tanzania

## Abstract

We assessed inpatient antibiotic use over six fiscal years at Tanzania’s national cardiac referral hospital. Overall use was 29.9 defined daily doses per 100 bed-days. Carbapenems were dominant. Reserve agents comprised 47.0%, and watch agents comprised 31.7%. Patterns indicate missed access targets and a need to strengthen stewardship in cardiac care.

## Introduction

Cardiovascular diseases are the leading cause of global mortality.^1^ Inpatient cardiac care frequently involves antibiotic exposure for peri-procedural prophylaxis and suspected infection. Monitoring antibiotic use is essential for stewardship, particularly in high-acuity low- and middle-income country settings, where empirical practice may accelerate resistance.^2^ Tanzania has national antibiotic consumption estimates from regulatory data sets.^3,4^ However, facility-level utilization in specialized cardiovascular settings remains sparse. The Jakaya Kikwete Cardiac Institute (JKCI) is Tanzania’s only dedicated cardiac referral center equipped with catheterization and open-heart surgical capabilities. We analyzed six fiscal years of inpatient dispensing records to describe antibiotic use patterns, the distribution of antibiotic classes, and the World Health Organization (WHO) Access–Watch–Reserve (AWaRe) categorization among cardiovascular inpatients.^5^

## Materials and methods

This retrospective longitudinal analysis used inpatient systemic antibiotic dispensing records from July 2016 to June 2022 at the JKCI (150 beds) in Dar es Salaam, Tanzania. Routine dispensing data were extracted from the MedPro system. Only systemic antibiotics (oral and parenteral) were included in this study.

Antibiotics were classified by anatomical therapeutic chemical (ATC) level 5 and by the 2021 WHO-AWaRe categories. Analyses were descriptive and inferential (SPSS v26); group differences in mean defined daily doses (DDD) per 100 bed-days were assessed using one-way ANOVA. There were no facility-specific antibiotic guidelines during the study period, and Tanzania’s National Antimicrobial Resistance National Action Plan was launched in 2017.^5^ Thus, no stewardship framework was yet operationalized at JKCI.

Antibiotic use was measured in DDD per 100 bed-days^6^ standardized to annual occupancy. An occupancy-corrected denominator was used to account for the bed count and fraction of each fiscal year in active clinical service using the formula :

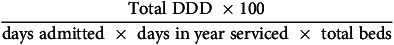




This adjustment was necessary because the JKCI was scaling up toward full capacity during its early stages of development. The fiscal years 2016–2017 had only 182.5 serviced days and 63 inpatients receiving antibiotics, explaining the lower numeric DDD values in the first year.

Expanded tables are available in the associated public preprint (medRxiv doi:10.1101/2025.09.24.25336595).

Ethical approval was obtained from the Muhimbili University of Health and Allied Sciences Research Ethics Committee and from JKCI. All data were anonymized and contained no identifiable patient information.

## Results

**Figure 1.**
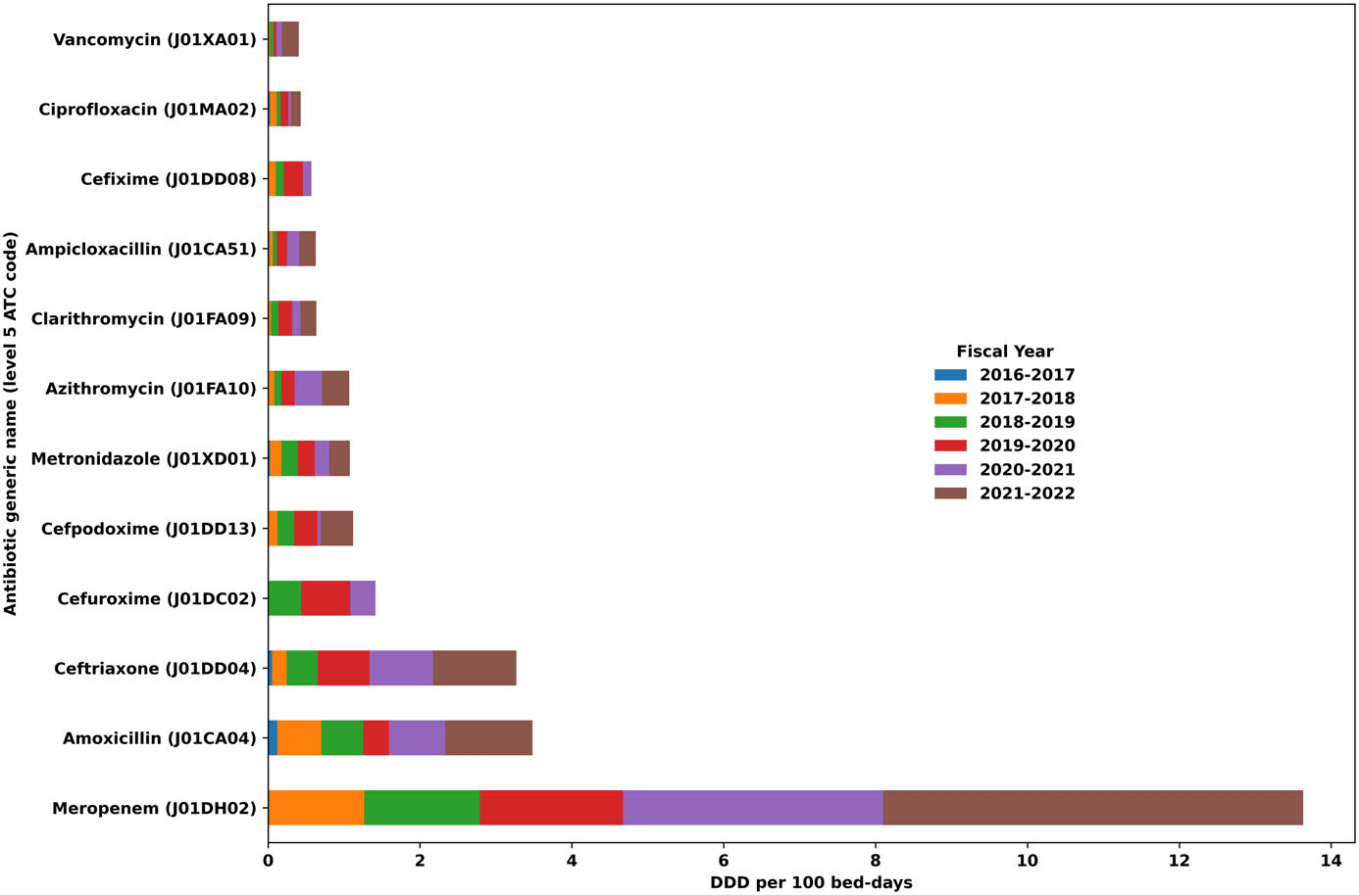
Top 12 Anatomical therapeutic chemical (ATC) level 5 antibiotic molecules contributing 91.4% of total inpatient use, expressed as defined daily doses (DDD) per 100 bed-days at Jakaya Kikwete Cardiac Institute, Tanzania, in 2016–2022.

A total of 30,885 inpatient antibiotic prescriptions from 6,612 cardiovascular inpatients were analyzed. Overall use was 29.88 DDD per 100 bed-days (Table 1). Carbapenems were the dominant ATC class and meropenem was the leading molecule (Figure 1). Reserve agents comprised 47.0% of all use (14.04 DDD/100 bed-days), Watch 31.7% (9.46) and Access 21.3% (6.38). Injectable formulations dominated (16.57 DDD per 100 bed-days; 55.5%), followed by tablets (9.59; 32.1%), syrups (2.06; 6.9%) and capsules (1.66; 5.6%).

**Table 1.** Patient characteristics and inpatients’ antibiotic use indicators at the Jakaya Kikwete Cardiac Institute, Tanzania, in 2016–2022. P-values indicate differences in mean defined daily doses (DDD) per 100 bed-days across categories within each variable, using ANOVA

Variable	Category	*N* (%)	Mean DDD per 100 bed-days (± SD)	Sum DDD/100	*p*-value
Sponsor group	AAR	19 (0.3%)	0.0099 (± 0.0159)	0.19	0.088
	Private	2004 (30.3%)	0.0043 (± 0.0170)	8.62	
	Exemption	39 (0.6%)	0.0028 (± 0.0042)	0.11	
	Military	64 (1.0%)	0.0126 (± 0.0706)	0.81	
	Jubilee insurance	17 (0.3%)	0.0071 (± 0.0105)	0.12	
	NHIF	4 249 (64.3%)	0.0045 (± 0.0189)	18.98	
	NSSF	67 (1.0%)	0.0038 (± 0.0065)	0.26	
	SIC insurance	40 (0.6%)	0.0035 (± 0.0046)	0.14	
	Zanzibar (SMZ)	96 (1.5%)	0.0064 (± 0.0183)	0.61	
	Other	17 (0.3%)	0.0022 (± 0.0037)	0.04	
Gender	Male	3 330 (50.4%)	0.0042 (± 0.0119)	13.93	0.154
	Female	3 282 (49.6%)	0.0049 (± 0.0246)	15.95	
Age group	0–9	1900 (28.7%)	0.0041 (± 0.0215)	7.74	0.161
	10–19	460 (7.0%)	0.0040 (± 0.0181)	1.85	
	20–29	320 (4.8%)	0.0030 (± 0.0063)	0.96	
	30–39	404 (6.1%)	0.0045 (± 0.0211)	1.8	
	40–49	487 (7.4%)	0.0040 (± 0.0123)	1.94	
	50–59	796 (12.0%)	0.0042 (± 0.0078)	3.36	
	60–69	1 124 (17.0%)	0.0050 (± 0.0192)	5.6	
	≥70	1 121 (17.0%)	0.0059 (± 0.0248)	6.64	
Fiscal year	2016–2017	165 (2.5%)	0.0045 (± 0.0096)	0.74	0.021
	2017–2018	1 141 (17.3%)	0.0042 (± 0.0259)	4.74	
	2018–2019	1 389 (21.0%)	0.0036 (± 0.0113)	4.95	
	2019–2020	1 282 (19.4%)	0.0042 (± 0.0191)	5.32	
	2020–2021	1 363 (20.6%)	0.0047 (± 0.0137)	6.34	
	2021–2022	1 272 (19.2%)	0.0061 (± 0.0246)	7.79	
Retention	Yes: started and ended in the same year	5 270 (79.7%)	0.0036 (± 0.0142)	10.69	<0.001
	No: continued into the following year(s)	1 342 (20.3%)	0.0080 (± 0.0319)	19.19	

## Discussion

Antibiotic use among cardiac inpatients was high and dominated by broad-spectrum and last-line agents. Carbapenems and third-generation cephalosporins accounted for most of the DDD volume. Similar patterns have been reported in African tertiary hospitals.^2,5^ The heavy reliance on injectables underscores opportunities for IV-to-oral switch, and the AWaRe imbalance—with Reserve and Watch accounting for 78.7% of all use—signals a clear stewardship priority.^7^ Empirical meropenem defaulting is probable in the absence of local guidelines and structured escalation pathways.^8^ Use of an occupancy-normalized denominator was essential for correctly interpreting the early partial year.^6^ Contextually, these findings sit within global increases in antibiotic consumption^9^ and global AMR burden estimates.^10^ JKCI therefore provides a benchmark for stewardship design in specialized cardiovascular inpatient care.

## Conclusion

Cardiovascular inpatients at Tanzania’s national cardiac center received substantial antibiotic exposure, dominated by Reserve and Watch antibiotics. Strengthening stewardship through AWaRe-aligned formulary oversight and IV-to-oral protocols can reduce unnecessary broad-spectrum use.
